# Effect of Packaging Method (Under Vacuum vs. Air) on Quality and Shelf Life of Carp (*Cyprinus caprio*) Fish Balls Stored at Fridge for 14 Days

**DOI:** 10.3390/molecules31040746

**Published:** 2026-02-22

**Authors:** Agnieszka Kaliniak-Dziura, Marek Kowalczyk, Monika Ziomek, Piotr Skałecki, Piotr Domaradzki, Ewa Poleszak, Jarosław Szponar, Mariusz Florek

**Affiliations:** 1 Department of Quality Assessment and Processing of Animal Products, University of Life Sciences in Lublin, Akademicka 13, 20-950 Lublin, Poland; piotr.skalecki@up.lublin.pl (P.S.); piotr.domaradzki@up.lublin.pl (P.D.); mariusz.florek@up.lublin.pl (M.F.); 2 Department of Food Hygiene of Animal Origin, University of Life Sciences in Lublin, Akademicka 12, 20-950 Lublin, Poland; monika.ziomek@up.lublin.pl; 3 Chair and Department of Applied and Social Pharmacy, Medical University of Lublin, Chodźki 1, 20-093 Lublin, Poland; ewa.poleszak@umlub.pl; 4 Clinical Department of Toxicology and Cardiology, Stefan Wyszynski Regional Specialist Hospital in Lublin, 100 Al. Kraśnicka, 20-550 Lublin, Poland; jaroslawszponar@umlub.pl; 5 Toxicology Clinic, Medical University of Lublin, 100 Al. Kraśnicka, 20-550 Lublin, Poland

**Keywords:** carp (*Cyprinus carpio*), fish balls, packaging, vacuum packaging, refrigerated storage, food quality, food shelf life, food safety

## Abstract

The study aimed to assess the influence of two packaging methods (under vacuum, VP vs. air, AP) on the quality of fish balls from carp (*Cyprinus carpio*) stored at +4 °C up to 14 days after preparation. The air-packed and vacuum-packed fish balls were analyzed for physicochemical parameters, microbiological status, and sensory characteristics. The packaging method and storage time interaction significantly (*p* < 0.05) affected the acid value (AV) and peroxide value (PV), as well as the thiobarbituric acid reactive substance index (TBARS), with lower values of these parameters observed in vacuum-packed samples at 7 d (AV, PV, TBARS), 9 d (TBARS), 12 d (PV) and 14 d (TBARS) of storage. Moreover, vacuum packaging helped maintain a beneficial oil absorption and pH, and partially slowed down the occurrence of undesirable changes in color, i.e., the decrease in redness of semi-raw fish balls or increase in yellowness of deep-fried products. Based on the overall quality values, the air-packed fish balls were sensory acceptable for up to 9 days, while the vacuum-packed fish balls were acceptable up to 12 d. The bacterial counts (total viable counts—TVC, psychrotrophic bacterial counts—PBC, total staphylococcal counts—TSC, sulfite-producing bacteria counts—SPBC, and lactic acid bacteria counts—LABC) increased during storage. Although the rate and pattern of growth varied depending on the packaging, fish balls maintained the recommended microbiological quality throughout the entire storage period. The VP method inhibited the growth of TVC, PBC, TSC, and SPBC relative to the AP method, while the VP method showed a higher increase in LABC. The results indicated that vacuum packaging appears to be an effective approach to prolong the shelf life of fish balls made from carp. Additionally, developing this convenient food product could be a valuable strategy to enhance consumer acceptance and promote the use of widely farmed carp species.

## 1. Introduction

Aquatic foods, especially those derived from aquatic animals, are valued as a rich source of high-quality protein, omega-3 fatty acids, minerals (iron, zinc, calcium, iodine) and vitamins (A, B_12_, D), considered key constituents of a nutritious diet [1]. In 2022, the global production of aquatic animals reached a record of 185 million tonnes (live weight equivalent), and, for the first time in the history, the farming of aquatic animals surpassed capture fisheries (51% of total production) [2].

The carp (*Cyprinus carpio*) is one of the most cultured freshwater fish species all over the world. The annual production exceeds 4 million tonnes, which qualifies *C. carpio* as one to the top ten species of aquatic animals produced [2]. Carp is also by far the most cultivated fish in Central and Eastern Europe pond aquaculture [3]. The high importance is probably due to its fast growth rate, easy cultivation and high feed efficiency ratio [4]. In Poland, one of the key European carp producers, fish farming has still retained its traditional character in terms of feeding or harvesting [5], and traditional carp ponds are an important part of human cultural heritage, with a high relevance for biodiversity [6]. Despite the high global production and great commercial importance of carp, consumers often considered this species as “too big” and difficult to prepare, with many bones and a moldy taste [7]. Therefore, it is difficult for carp to compete with highly processed and much more convenient fish products made from different fish species. To increase the attractiveness and demand for carp products, many processors search for alternatives and extend the range of carp products [8].

Fish balls are defined as a spherical fish product made by mixing fish flesh with starch, seasonings and vegetables [9]. This fish product is characterized by its relatively low cost, convenient eating properties, and high nutritional value [10,11].

Fish balls, however, are mainly popular in Asia—used as an addition to soups or served fried, boiled, or steamed [12], often by street food restaurants. In Europe, fish balls are not particularly popular, at least not in the form in which they are commonly found in Asia. Moreover, a high level of moisture and protein makes fish balls highly perishable, with a relatively short shelf life of 4–5 days stored at approx. +5 °C and 2 days stored without refrigeration [13]. Although freezing fish balls enables them to last longer, consumers and retailers might not prefer this method of preservation, as it tends to affect the texture and freshness of the fish balls [14]. Therefore, it is decisive to extend the shelf life of fish balls, maintaining their high sensory and nutritional values.

Extending the shelf life of food and fishery products can be achieved through the combination of food packaging and low-temperature storage [15]. Vacuum packaging provides an anaerobic environment with good barrier properties towards air and water, inhibiting the growth of aerobic spoilage microorganisms like, e.g., *Pseudomonas* spp. and limiting oxidative rancidity [15]. Therefore, vacuum packaging, as the one of the natural preservation packaging methods, can effectively maintain not only the shelf life but also the overall quality of muscle foods for a long time [16]. Studies on sensory and microbiological analysis [14] have shown that vacuum packaging may extend the shelf life of fish balls by 6 days compared to air packaging (<3 days).

Unfortunately, the number of papers on *C. carpio* products is very limited. Moreover, in the available scientific literature, there are no existing studies conducted on fish balls prepared from the meat of fish kept in Europe. Carp reared in different regions (e.g., in Asia) often experience different aquaculture practices and environmental conditions. In the classical Central–Eastern European production system, carp is a dominant species. It belongs to the tertiary trophic level and consumes zooplankton and zoobenthos from the secondary trophic level. In the multispecies cultures of Asia, which are economically more productive, carp is stocked together with herbivorous fishes. The Asian fish species of the second trophic level consume algae from the primary production level and higher water vegetation as well [17]. The genome sequencing of European and Asian domestic common carp strains showed that they even formed two distinct clades as a result of their diverse geographical habitats and domestication histories [18]. It is known that carp’s composition, which can affect its shelf life (such as the content of pigments, fatty acids, or the activity of endogenous enzymes), depends on genetics and diet [19,20]. However, there are no existing reports on the comprehensive comparative analysis of the composition of *Cyprinus carpio* carp reared in Europe and other regions. In consequence, it is difficult to predict how fish balls prepared from *C. carpio* cultivated in Europe will behave during refrigerated storage and what their shelf life will be.

Fish balls prepared from *C. carpio*, with its high protein and low fat content, may effectively fulfill the demands of nutritional diets for today’s consumer. In particular, frying the fish ball achieves an excellent brown color and desirable flavor, which attracts consumer acceptance worldwide. Moreover, fish protein is easily digested, which makes this product adequate even in the diet of patients and growing children [21]. In the European food market, fish balls may represent a novelty, with a high chance of meeting the interest of consumers seeking convenient food solutions. Moreover, taking into account the clear trend toward low-processed foods with short ingredient lists, products with ultra-short formulations (e.g., consisting solely of fish meat, salt, and pepper) are likely to attract considerable consumer interest.

Taking into account the above points, research was carried out to develop fish balls with a very simple ingredient composition using minced meat from European-farmed carp *Cyprinus carpio* and to thoroughly examine their physicochemical, texture and color parameters, hydrolytic and oxidative stability, and sensory and microbiological properties during a 14-day period of refrigerated storage (+4 °C) under air (AP) and vacuum packaging (VP). This study provides information that could be useful in developing convenient food products from *Cyprinus carpio* cultured in Europe, which could promote a better utilization of cyprinid species and contribute to more sustainable aquaculture practices.

## 2. Results and Discussion

### 2.1. Chemical Composition and Partial Sums of Fatty Acids of Raw Meat and Fish Balls (Semi-Raw and Deep-Fried)

The chemical composition of 100 g of raw carp (*Cyprinus carpio*) meat, semi-raw, and deep-fried fish balls is presented in Table 1. The results for raw carp meat were consistent with the existing data for farmed carp in Europe [22,23]. In turn, compared to the chemical composition of Asian carp reported by Li et al. [24] (moisture 78.93 ± 2.82%, ash 1.01 ± 0.06%, protein 15.39 ± 1.29%, and lipids 0.96 ± 0.45%), the analzyed fish balls were characterized by a lower moisture content and higher levels of protein and lipids. The preaparation significantly (*p* < 0.05) affected the chemical composition of carp meat. Semi-raw fish balls were characterized by a significantly (*p* < 0.05) lower moisture content and significantly (*p* < 0.05) higher protein and ash contents compared to raw carp meat, and these differences probably resulted from water release that occurred during manual fish ball formation or pre-microwave treatment. Huda et al. [25], for Malaysian fish balls, reported a higher level of moisture (72.5–89.9%) and lower level of protein (7.54–9.89%), lipids (0.13–1.75%), and ash content (1.61–3.18%) compared to those observed in our study. Significant (*p* < 0.05) changes in proximate compostion were also observed after the deep-frying process. In this case, the moisture content in deep-fried fish balls decreased and the protein, lipid, and ash level incerased, compared to the semi-raw samples. These changes were most likely the result of water loss and the absorption of vegetable oil during frying. In addition, the increase in ash content might be partly due to the addition of NaCl and pepper. Ivanova et al. [22] reported a similar trend for the chemical composition of fish steak fried in oil.

The partial sums of fatty acids (% of total fatty acids) in raw carp meat, semi-raw and deep-fried fish balls are presented in Table 1. The raw carp meat was characterized with a dominant proportion of monounsaturated (MUFA), followed by saturated (SFA) and polyunsaturated fatty acids (PUFA). Such proportions (MUFA > SFA > PUFA) were also reported by other authors [22,26,27] for *C. carpio* farmed in Europe. In turn, a study performed on five common carp strains reared in China observed the following proportions of fatty acids: MUFA > PUFA > SFA [28]. In Turkey, where carp is mainly caught from inland waters, PUFA (29.3–42.8%) can be found higher than MUFA (28.3–41.1%) or SFA (26.6–29.6%) [29]. Nevertheless, the preparation step and application of heat treatment significantly affected the fatty acid profile of carp meat (Table 1). The semi-raw fish balls were characterized by significantly lower MUFA but higher PUFA compared to raw meat, which was likely due to the manual forming of the product or pre-microwaving applied in the product preparation step. Heat treatment may influence the composition of fatty acids in fish, depending on the temperature, contact surface width, fish size and initial fat content [30]. In this case, the lower SFA and higher MUFA and PUFA shares in deep-fried fish balls compared to semi-raw samples were probably the result of deep-frying and the incorporation of PUFA present in the cooking oil. The increase in PUFA and the decrease in SFA after thermal treatment have previously been reported for products obtained from farmed common carp (*Cyprinus carpio*) [22].

### 2.2. Physicochemical Properties of Semi-Raw Fish Balls

The initial pH of semi-raw fish balls was 6.81 (Table 2 and Figure S1). The statistical analysis revealed that the pH was significantly affected by the packaging method, storage day, and the interaction between these factors. The pH of air-packed fish balls exhibited a significant fluctuation during subsequent storage days. Initially, a significant (*p* < 0.05) decline in comparison to the control value (0 d) was observed at 2 d of storage, then the pH gradually (*p* < 0.05) increased up to 7 d, followed by a further significant (*p* < 0.05) decrease at the end of the storage at 14 d. The initial decrease in pH in air-packed samples might be attributed to the accumulation of lactic acid produced through glycolysis [31]. In turn, the subsequent increase followed by a decrease in pH was similar to the results revealed by Chen et al. [10] for the fish balls of *Ctenopharyngodon idellus* stored in air in refrigerated conditions. They attributed the described changes to the generation of basic nitrogen compounds (early stages of storage) as well as to the oxidative rancidity of fat and the activity of indigenous lactic acid bacteria that metabolize lactose to lactate (later stages of storage). In contrast to the AP group, the pH of vacuum-packed fish balls was maintained at a relatively stable range (between 6.79 and 6.84), with no significant differences compared to the control value (0 d).

The storage time and packaging method as well as the storage time × packaging method interaction had no significant effects on the water activity of semi-raw fish balls, as expected. The a_w_ values of fish balls ranged between 0.86 and 0.88. In freshly caught fish, including carp, a_w_ is usually above 0.95 [32,33]. Nevertheless, the addition of salt decreases the a_w_ value and, as a consequence of the restriction of the available water, can also inhibit microbial growth [34]. Moreover, during the refrigerated storage, food products packed in air may dry out, which additionally decreases the a_w_ values. Still, fish balls are usually considered as products with a high water activity and are prone to the growth of microorganisms [13], and the analyzed products also must be classified as a moist food (a_w_ > 0.85) [32].

The OA was significantly (*p* < 0.05) affected by the packaging method and day of storage. Both packaging methods significantly (*p* < 0.05) increased the percentage of oil absorption with storage time (Table 2 and Figure S1). Compared to the control value (0 d), the OA in all samples during subsequent experimental days was significantly (*p* < 0.05) higher. Moreover, in the present study, vacuum-packed samples were characterized by significantly (*p* < 0.05) lower levels of oil absorption (on average 31.38 ± 2.19%) compared to air-packed products (on average 33.09 ± 2.69%). Although the oil absorption may depend on many factors (e.g., oil quality, frying time, oil and food temperature, etc.) [35,36], the differences observed in our study were probably due to the changes that occur during the vacuum packaging process. The pressure may cause liquid exudation [37], and food with a lower moisture content usually represents a lower oil uptake [38]. Moreover, vacuum packaging may inhibit the growth of mesophilic aerobic microorganisms [39], which are responsible for destroying product structure and integrity and, consequently, the increase in oil absorption in the product. This may also explain the lower values of OA in the VP group compared to the air-packed samples. The OA in the analyzed samples varied from 27.18% to 35.61%, and these values were within the range typical for fried food products (6–40%) [40]. A similar oil absorption (between 30.00% and 31.67%) was earlier observed by Fofandi et al. [36] in battered and breaded filets of *Aluterus monoceros* fried in similar conditions (temp. 150–170 °C, time 8–12 min).

### 2.3. Texture Parameters of Semi-Raw Fish Balls

An important effect of the interaction of storage time x packaging method on the shear force of semi-raw fish balls was observed. The WBSF of air-packed samples decreased with storage time, and the shear force observed at 14 d was significantly (*p* < 0.05) lower compared to the level recorded at 2 d (Table 2 and Figure S1). The shear force test is widely used to evaluate the meat tenderization degree in *post mortem* muscle [41], with lower values indicating more tender meat [42]. Texture loss during storage in chilling conditions has been previously reported in fish flesh [43,44,45,46], as well as in fish products, including fish balls [9,47]. The tenderization of fish during storage is probably due to the proteolysis induced by endogenous cathepsins and microbial activity [45] or protein oxidation that may also have a negative impact on the tenderness of muscle food [48]. The WBSF value of vacuum-packed samples showed a fluctuating trend during cold storage, with the lowest value at 7 d; however, significant differences were not observed between days. A similar variation trend in hardness (N) was observed by Noordin et al. [14] during the refrigeration storage of air- and vacuum-packed cooked fish balls. The shear energy of semi-raw fish balls was not affected by any factors and had no trend pattern, ranging from 439.28 mJ to 821.95 mJ during storage (Table 2 and Figure S1). Therefore, this texture parameter was not sensitive to changes in texture with storage time. Similar results were previously reported by Bainy et al. [49] during the frozen storage of tilapia burgers.

### 2.4. Color Parameters of Semi-Raw and Deep-Fried (Internal and External Surface) Fish Balls

The color of semi-raw carp (*Cyprinus carpio*) fish balls stored at +4 °C under air and vacuum conditions is shown in Table 3 and Figure S2. In the cases of lightness (L*) and redness (a*) indices, the significant (*p* < 0.05) effects of the storage time, packaging, and storage time x packaging interaction were observed, whereas the value of yellowness (b*) was significantly (*p* < 0.05) differentiated only by the packaging method. The initial values of L*, a* and b* were similar to the ranges reported by Huda et al. [25] for commercial Malaysian fish balls (L* between 69.61 and 77.96, a* between 2.02 and 0.33, b* between 15.66 and 19.70) and by Nuryahyani et al. [50] for fish balls of starry trigger (*Abalistes stellaris*) (L* 68.54 ± 0.40, a* 2.97 ± 0.19, b* 15.05 ± 0.95). The lightness of air-packed fish balls showed a fluctuating trend during refrigerated storage, with the highest levels at 5 d and 12 d. Samples packed in air were lighter compared to products packed in a vacuum; however, significant (*p* < 0.05) differences were recorded only at 5, 9 and 12 d. In vacuum-packed products, the lightness was at a stable level up to 12 d, then a significant (*p* < 0.05) increase at 14 d was observed. White fish balls are generally more preferred by consumers, as it indicates the freshness of the product [25]. Lipid oxidation and protein decomposition, which occur with prolonged storage as a result of microorganism multiplication and enzyme activity, lead to a deterioration in the quality of fish products and decrease in lightness [51]. In the present study, the L* value did not decline below the initial value (0 d). Therefore, the brightness of analyzed products was expected to be positively perceived by consumers over 14 days of refrigerated storage.

The redness of air-packed fish balls decreased significantly (*p* < 0.05) with storage time, whereas, in vacuum-packed samples, it increased significantly (*p* < 0.05) at 5 d, and then remained at a similar level up to the end of the storage. Throughout the whole storage period, the samples packed in air were significantly (*p* < 0.05) less red, compared to the fish balls packed in a vacuum, assuming negative values at 12 d and 14 d, which represent blue. The redness of muscle is generally proportional to the myoglobin concentration and to alterations in the protein and redox state of the heme iron. Microbial spoilage and chemical changes such as the oxidation of lipids and proteins result in the autoxidation of myoglobin, especially the conversion of the bright red oxy-myoglobin to the brownish met-myoglobin form, which is mainly responsible for the loss of redness color during storage [52]. This may explain the decrease in the a* value observed in air-packed fish balls and its lower level compared to vacuum-packed samples.

The yellowness of fish balls from the VP group were significantly (*p* < 0.05) higher compared to the AP group, although the numerical differences were not large (approximately 0.2 units). Throughout the storage period, air-packed fish balls differed significantly (*p* < 0.05) in color (all coordinates) in comparison with the samples before packaging, whereas in vacuum-packed samples significant (*p* < 0.05) differences (in comparison to 0 d) were observed for b* and L* (14 d). This may suggest that vacuum packaging could partially slow down the occurrence of undesirable changes in color, i.e., the decrease in redness of semi-raw fish balls stored at +4 °C.

Analyzing the values of total color difference (∆E), it is commonly accepted that, if the samples are not next to each other, a total color difference of less than 1 is imperceptible. In turn, Frances and Clydesdale [53] reported that the value ΔE = 2 can already be perceptible in the visual assessment of many products. From a technical point of view, the color differences (in CIE L*a*b* units) between 1.1 and 2.8 align with rigorous tolerances, and those between 2.8 and 5.6 align with normal tolerances, while values over 5.6 ought to be easily distinguished by the human eye [54]. Therefore, it can be concluded that the differences in the color of the semi-raw fish balls during storage might not be noticeable by consumers when compared to 0 d.

In terms of the external surface of deep-fried fish balls, the significant (*p* < 0.05) effect of the storage x packaging interaction was observed for a* and b* coordinates, whereas the lightness was significantly (*p* < 0.05) affected by the storage time. Starting from 2 d (56.21 ± 2.19), the L* values gradually decreased with the storage time, and significant (*p* < 0.05) differences were found between 12 d (53.24 ± 1.61) and 14 d (51.36 ± 2.13) (Table 3 and Figure S3). The gradual darkening of the fish balls might be attributed to the decreases in the substrates for nonenzymatic browning reactions, particularly carbohydrates, with the increasing storage time [55]. The color of thermally processed seafood products is a very important quality factor that influences the initial acceptance of the consumer [56]. The ideal color for fried products was generally reported to be “light brown” [57] or “light golden” [58]. The frying process causes a decrease in the lightness and increase in the redness and yellowness in fish products in relation to the raw sample [58,59]. These color changes are due to the loss of water and the absorption of oil through heat and mass exchange. The product becomes crusty as a result of the loss of water and acquires a light brown color due to Maillard’s reactions, which occur between reducing sugars (glucose and fructose) and amino acids (asparagine) at high temperatures [60]. The color of the external surface of deep-fried fish balls (Table 3) was similar to the results reported by Zeng et al. [61] for the crust of deep-fried battered and breaded fish balls prepared from frozen silver carp surimi, except for the lower L* values (L* 60.53 ± 0.06, a* 9.31 ± 1.43, b* 26.29 ± 0.07). Similarly, Omidiran et al. [62] reported color values for deep-fried fish nuggets that were comparable to those obtained in our study (L* 25.72–89.60, a* 7.29–28.33, b* 33.50–58.64). The share of redness of both air- and vacuum-packed fish balls increased significantly (*p* < 0.05) with the storage time; however, the samples packed in a vacuum were less red compared to the samples packed in air, with significant (*p* < 0.05) differences observed from 5 d to 14 d. The yellowness of fish balls packed in air decreased at 5 d and remained at a similar level until day 14, when a significant (*p* < 0.05) increase was observed. In vacuum-packed fish balls, the b* value decreased gradually (*p* < 0.05) up to 7 d, remained at a similar level at 9 d and 12 d, and then increased significantly (*p* < 0.05) at 14 d. The b* value of samples packed in a vacuum at 7 d, 9 d and 14 d was significantly (*p* < 0.05) lower than in air-packed samples. A similar decrease in lightness and an increase in a* and b* coordinates were observed earlier by Turkkan et al. [63] in vacuum-packed fried sea bass stored for 15 days at 2 °C. Other authors reported that the different pattern changes in color parameters observed during the storage of fish products may differ depending on different species, product types or storage conditions [55,64]. The fish balls of the AP and VP groups differed significantly in lightness (from 7 d) and redness (throughout the storage time) compared to the samples at 0 d. In the case of the b* coordinate, significant (*p* < 0.05) differences compared to 0 d were observed at 2 d and 14 d in fish balls packed in air, as well as at 2 d, 7 d, 9 d and 14 d in vacuum-packed samples. As the ∆E for the external surface of deep-fried fish balls at most sampling days was between 2.8 and 5.6, the color differences in the stored samples when compared to 0 days may be noticeable to the consumer.

The color of the internal surface of deep-fried carp fish balls stored at 4 °C under air and vacuum conditions is shown in Table 3 and Figure S4. A significant (*p* < 0.05) effect of storage time was found for all analyzed color indices, whereas the significant (*p* < 0.05) storage time x packaging interaction was limited to yellowness. No effect for the packaging method was observed. The lightness of fish balls decreased with the storage time, with significantly (*p* < 0.05) lower values at 14 d (61.79 ± 3.74) compared to 5 d, 7 d and 9 d (65.72 ± 2.51, 65.70 ± 3.22, 65.79 ± 2.91, respectively). In contrast, the redness was highest at the end of storage (2.06 ± 0.97 at 14 d), differing significantly (*p* < 0.05) from the values at 2 d (0.99 ± 0.28), 9 d (1.25 ± 0.28) and 12 d (1.14 ± 0.49). The changes in L* and a* corresponded with the results obtained for the product external surface (Table 3). Our results were also consistent with those reported by Zeng et al. [61] for the core of silver carp surimi fish balls (L* 73.38 ± 1.46, a* 0.56 ± 0.38, b* 15.46 ± 0.48), although the L* values in our study were lower, and with the values reported by Turkkan et al. [63] for fish products prepared from fried sea bass stored at refrigerated conditions for 15 days. In the present study, the level of yellowness in both groups of fish balls remained at a similar level up to 12 d; then, a significant (*p* < 0.05) increase in b* was observed at 14 d. The increase in yellowness during storage was probably due to the accumulation of secondary lipid oxidation products and the products of the interaction between aldehyde generated during the oxidation of lipids and the amine groups of phospholipids [65]. The positive correlation between increasing lipid oxidation and instrumental yellowness (b*) has been previously described in fish [66] and seafood [65]. This may also explain why in air-packed samples the final yellowness (14 d) was significantly (*p* < 0.05) higher compared to the values recorded at 2 d, 5 d, 7 d, 9 d and 12 d, whereas the vacuum-packed samples differ significantly (*p* < 0.05) only with the value at 12 d. Similarly, the significantly (*p* < 0.05) lower yellowness in the samples packed in a vacuum, compared to products packed in air, was probably the result of the vacuum packaging, which limits the access of oxygen and may therefore slow down the oxidation processes and development of yellow pigmentation. At the end of storage (14 d), the air-packed fish balls differed significantly (*p* < 0.05) in terms of a* and b* indices from the values obtained at 0 d, whereas vacuum-packed samples were characterized by significantly (*p* < 0.05) higher values of L* and b* compared to 0 d. Taking into account the values of ∆E obtained after frying, the color of the internal surface of fish balls may differ noticeably during storage when compared to the 0 d samples.

### 2.5. Sensory Properties of Deep-Fried Fish Balls

In the present study, no significant effects of storage time or/and packaging method were observed in terms of most sensory attributes (Table 4). As is commonly known, the organoleptic properties of fish balls change during the storage process, due to the external effects of microorganism activity and chemical reactions of food ingredients [10]. The vacuum-packed fish balls were considered by the panelists to be significantly (*p* < 0.05) more juicy compared to the air-packed ones throughout the storage duration. However, in both analyzed groups of fish balls, the juiciness increased up to 9 d, then decreased gradually to the end of the storage period.

Finally, the overall quality of fish balls packed in a vacuum was scored significantly (*p* < 0.05) higher (6.8 ± 1.5) than products packed in air (6.2 ± 2.1). High scores (>5) were given up to 9 d and 12 d in the AP and VP groups, respectively; after that, the overall quality decreased until to the end of storage. The scores below 5 recorded at 12 d and 14 d for fish balls packed in air and at 14 d for samples packed in a vacuum were significantly (*p* < 0.05) lower compared to 0 d, suggesting that the products might not be acceptable for consumption. This finding is in agreement with Akkuş et al. [67], who reported a shelf life of approximately 9 days for refrigerated raw and boiled fish balls prepared from anchovies (*Engraulis encrasicholus*). In turn, Chen et al. [10] revealed that the shelf life of fish balls produced from *Ctenopharyngodon idellus* stored under refrigerated conditions was approximately 12 days; however, a deterioration of sensory characteristics was observed already at 10 d. Also, Noordin et al. [14] reported that vacuum packaging extended the shelf life of fish balls by 6 days compared with air packaging (<9 days). This was longer (by 3 days) than in our study; however, the cited authors assessed fish balls at longer intervals and did not analyze the samples at 12 d.

### 2.6. Hydrolytic and Oxidative Stability of Semi-Raw Fish Balls

**Figure 1:**
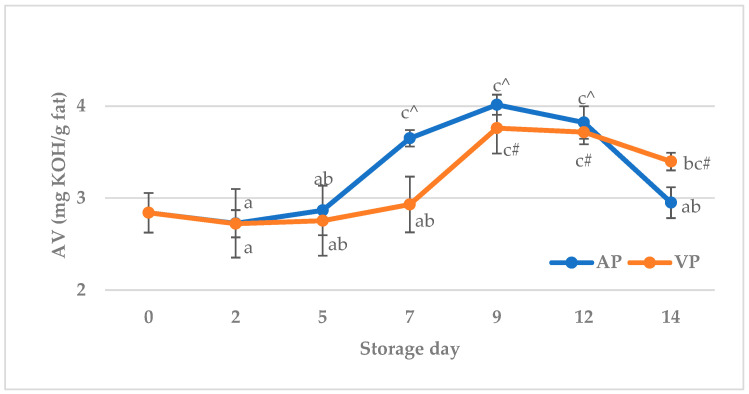
The acid value (AV) of carp (*Cyprinus carpio*) fish balls stored at +4 °C under air (AP) and vacuum (VP). a, b, c—Means with different superscripts are significantly different (*p* < 0.05); ^—means with this symbol under the air packaging method are significantly different from day 0 (*p* < 0.05); #—means with this symbol under the vacuum pressure method are significantly different from day 0 (*p* < 0.05).

The acid value of semi-raw carp fish balls is illustrated in Figure 1. The initial AV was found to be 2.8 ± 0.2 mg KOH/g of fat, which was similar to cyprinid filets (2.93 ± 0.16 mg KOH/g of fat), reported by Skałecki et al. [68]. Significant (*p* < 0.05) effects of storage time and storage x packaging interaction on the AV were recorded. In both packaging methods, the level of AV increased up to 9 d, then decreased to the end of the storage period. Significant (*p* < 0.05) differences were observed between day 5 and 7 in air-packed fish balls and between day 7 and 9 in vacuum-packed samples. The decrease recorded between 12 and 14 d in the AP group was significant (*p* < 0.05). An increase in AV is generally associated with lipase activity originating from microorganisms or animal tissues [69]. Aubourg et al. [70] revealed that the formation of free fatty acids during the first stages of the chilling treatment (up to day 9, approximately) is usually the result of the activity of endogenous enzymes (lipases and phospholipases), while in the later stages these compounds are mostly produced as a result of bacterial catabolic processes. The present results for AV may then suggest that the enzyme activity was considerable, while the contribution of microorganisms’ activity to lipid hydrolysis in the period from 11 d to 14 d was minimal, and this might be the result of salt addition, with its inhibitory effect on lipid hydrolysis development in stored fish [71,72]. In addition, the depletion of substrates or oxidation of free fatty acids could also be responsible for the decrease in AV after 9 d [73]. Compared to samples packed in air, the acid value of fish balls packed in a vacuum was lower up to 12 d; however, a significant (*p* < 0.05) difference was confirmed only at 7 d. The AV recorded at 7, 9, and 12 d in air-packed fish balls and at 9, 12, and 14 d in vacuum-packed samples was significantly (*p* < 0.05) higher compared to the initial acid value. According to EFSA [74] and CAC [75] guidelines, the AV should not exceed 3 mg KOH/g; however, the same sources state that the acid value of fish oils with a low content of free fatty acids is typically in the range from 0 to 5 mg KOH/g. It can therefore be concluded that, up to 5 d (AP group) or 7 d (VP group), the tested fish balls met the strict standards regarding the acid value (≤ 3 mg KOH/g), and even the later stages of storage hydrolytic rancidity should not have had a negative impact on the product quality and safety.

**Figure 2:**
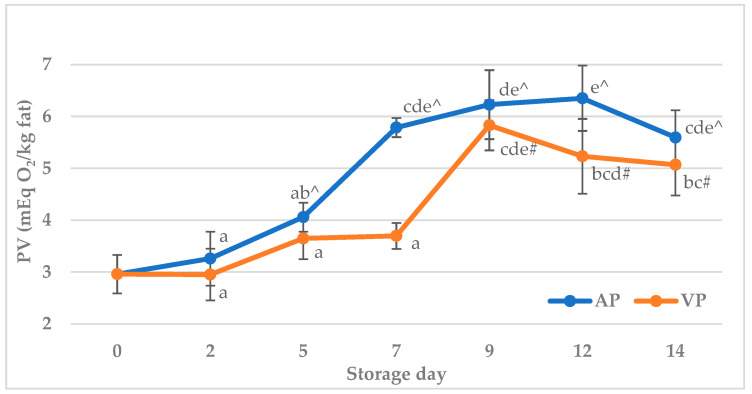
The peroxide value (PV) of carp (*Cyprinus carpio*) fish balls stored at +4 °C under air (AP) and vacuum (VP). a, b, c, d, e—Means with different superscripts are significantly different (*p* < 0.05); ^—means with this symbol under the air packaging method are significantly different from day 0 (*p* < 0.05); #—means with this symbol under the vacuum pressure method are significantly different from day 0 (*p* < 0.05).

The peroxide value of semi-raw carp (*Cyprinus carpio*) fish balls is presented in Figure 2. The level of the PV was significantly (*p* < 0.05) differentiated by the storage time and packaging method, as well as the storage time x packaging method interaction. The initial PV was 3.0 mEq O_2_/kg fat. With storage time, the PV increased significantly (*p* < 0.05), reaching a maximum level in air-packed samples at 12 d (6.4 mEq O_2_/kg fat) and in vacuum-packed samples at 9 d (5.8 mEq O_2_/kg fat), and then started to decrease. The PVs obtained in the VP group were lower compared to the samples packed in air; however, significant (*p* < 0.05) differences were recorded only at 7 d and 12 d. After 5 d for air-packed samples and 9 d for samples packed in a vacuum, the PVs were significantly (*p* < 0.05) higher compared to the control value (0 d). While the significant formation of primary oxidation products in air-packed samples can be explained by the presence of oxygen, the reason for the increasing trend in the peroxide value observed in vacuum-packed fish balls is not entirely clear. It is most likely related to the muscle tissue prooxidant system, particularly lipoxygenase. Lipoxygenase is a major enzymatic initiator of lipid oxidation in fish tissues and is capable of directly oxygenating polyunsaturated fatty acids (PUFAs), even when they are present in phospholipids bound to cell membranes, leading to the formation of lipid hydroperoxides. Therefore, this enzyme may be involved in the initiation of lipid oxidation in fish muscle even under vacuum packaging conditions [76]. In addition, the free ionic iron released from heme pigments and ferritin act as a major catalyst for lipid peroxidation in raw and cooked meat [77]. Heme-initiated lipid peroxidation is autocatalytic and also forms lipid hydroperoxides [78]. This may explain the increase in the peroxide value at the beginning of the storage observed in vacuum-packed fish balls. Another possible explanation for these changes may be the imperfection of the vacuum packaging system. The vacuum does not guarantee a complete removal of residual oxygen, as some O_2_ may remain trapped within the food matrix or the packaging material. Moreover, additional oxygen can permeate from the air atmosphere into the packaging material during product storage depending on the barrier properties of the packaging material [79]. Nevertheless, even the maximum level of PV observed in this study did not exceed the acceptable range of 10–20 mEq O_2_/kg fat, when the rancid flavor is perceptible [80]. Moreover, the result of this study is also in accordance with the findings of earlier investigations [47,81,82], where a similar increasing effect of storage conditions on the PV for refrigerated fish products was found. For instance, Singh et al. [47] reported that the PV of ready-to-eat fish balls prepared from Rohu (*Labeo rohita*) mince increased from an initial value of 1.52 mEq O_2_/kg fat to 3.72 mEq O_2_/kg fat at the end of 28 days of storage at 4 ± 1 °C under aerobic packaging. Also, Salman et al. [81] documented a significant increase in the PV (from 3.8 meq to 16.6 mEq O_2_/kg fat) for silver carp (*Hypophthalmichthys molitrix*) cutlets over 16 days of refrigerated storage. In turn, Vanitha et al. [82] reported that, in a fresh catla (*Catla catla*) fish burger, the PV was 4.62 mEq O_2_/kg, but increased to 7.28 mEq O_2_/kg and then decreased to 4.98 mEq O_2_/kg at 17 d. This decrease in the PV at the end of storage may occur due to the decomposition of hydroperoxides into secondary oxidation products, which was also observed in the present study.

**Figure 3:**
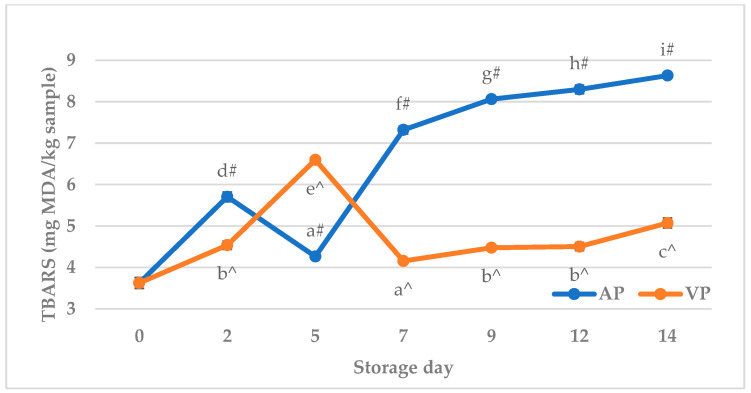
The thiobarbituric acid reactive substances (TBARS) of carp (*Cyprinus carpio*) fish balls stored at +4 °C under air (AP) and vacuum (VP). a, b, c, d, e, f, g, h, i—Means with different superscripts are significantly different (*p* < 0.05); ^—means with this symbol under the air packaging method are significantly different from day 0 (*p* < 0.05); #—Means with this symbol under the vacuum pressure method are statistically different from day 0 (*p* < 0.05).

The TBARS of semi-raw carp (*Cyprinus carpio*) fish balls is presented in Figure 3. The TBARS value was significantly (*p* < 0.05) affected by the storage time and packaging method, as well as the storage time x packaging method interaction. The TBARS of air-packed fish balls initially increased significantly (*p* < 0.05) at 2 d, then decreased (*p* < 0.05) at 5 d, and subsequently increased (*p* < 0.05) until the end of the storage period. In vacuum-packed fish balls, the TBARS significantly (*p* < 0.05) increased up to 5 d, then decreased (*p* < 0.05) at 7 d, and subsequently increased (*p* < 0.05) until the end of the storage period. Throughout the entire storage period, the TBARS of all packed samples differed significantly compared to the initial value (3.1 ± 0.1 mg MDA/kg product). The values obtained in the VP group from 7 d were, however, significantly (*p* < 0.05) lower compared to the AP group. The increase in the TBARS with the storage time in refrigeration conditions has been previously recognized both in fish filets [44,83] and in fish products [9,10,82]. It has been suggested that a maximum TBARS value, indicating the good quality of the fish, is 5 mg MDA/kg product, while fish may be consumed up to a TBARS value of 8 mg MDA/kg product [84]. The obtained results may suggest that the oxidative rancidity in vacuum-packed samples remained relatively low throughout the entire period of storage at +4 °C, and this level was within the acceptability limits for fish consumption. In air-packed products, the TBARS values at 9 d and 12 d were close to 8 MDA/kg product and exceeded the indicated limit at 14 d. This is in accordance with the results reported by Chen et al. [10], where the most rapid increase in TBARS in air-stored fish balls occurred after 8 day of refrigerated storage. Although the present study did not confirm direct trends for the TBARS index, its fluctuations were also previously observed by Arashisar et al. [83] in air- and vacuum-packed rainbow trout filets stored in chilling conditions. The initial peak in TBARS observed in both air- and vacuum-packed samples was most likely due to the non-specific nature of the TBARS assay. Thiobarbituric acid (TBA) can react not only with malondialdehyde (MDA) but also with other aldehydes, protein and sugar degradation products, as well as with amino acids and nucleic acids [85,86]. On the other hand, as in the case of primary lipid oxidation products, the influence of the muscle tissue prooxidant system on the TBARS value cannot be excluded. Heme iron promotes the propagation of lipid oxidation by decomposing pre-formed peroxides, e.g., hydrogen peroxide and lipid hydroperoxides, into free radicals via Fenton-like reactions [78,87]. As a consequence, the hydroperoxides are further decomposed to flavorful secondary oxidation products, mainly aldehydes, such as hexanal, 4-hydroxynonenal, and malondialdehyde [88], which may lead to the increase in TBARS even in vacuum-packed fish balls. In this context, the pre-microwaving step applied in this study during the fish ball preparation may also influence the TBARS level. This is because the heat treatment is known to accelerate lipid oxidation and volatile compound formation in meat by disrupting muscle cell structure, inactivating antioxidant enzymes and other antioxidant compounds, and promoting the release of iron from heme pigments [76]. Finally, in addition to the mechanisms discussed above, the TBARS level may also be influenced by the instability of secondary lipid oxidation products, which may decompose or enter into interactions in which MDA and hexanal form adducts with lysine residues in meat proteins [84,89,90]. Thus, this may also explain the TBARS fluctuation observed in this study.

### 2.7. Microbiological Stability of Semi-Raw Fish Balls

The microbiological analysis showed that bacterial counts in all tested groups (TVC, PBC, TSC, SPBC, LABC) increased during storage, although the rate and pattern of growth varied depending on the packaging method (Table 5). The vacuum packaging reduced the growth of TVC, PBC, TSC, and SPBC compared with air packaging, while LABC showed a greater increase in vacuum-packed samples. According to Özpolat et al. [91], a similar inhibitory effect on the growth of aerobic bacteria was observed, suggesting a possible delay of spoilage processes. According to European Commission Regulation No 2073/2005 [92], food safety criteria for fishery products require the absence of *Salmonella* spp. in 25 g of product, while the criterion for *Listeria monocytogenes* (≤100 CFU/g or absence in 25 g before distribution) applies only to ready-to-eat foods. As the tested carp fish balls were not ready-to-eat (non-RTE), the *Listeria monocytogenes* requirement does not apply. Although no specific process hygiene criteria have been established for fishery products, European Commission Regulation No 852/2004 [93] obliges producers to maintain microbiological quality through proper hygiene and process control. In such cases, a total viable count—TVC—below 6 log CFU/g and *Enterobacteriaceae* below 2 log CFU/g are commonly used to assess hygiene and product stability. It should also be emphasized that microwave treatment can contribute to a reduction in the initial microbial load and influences the growth dynamics of the surviving bacteria during storage [94,95,96]. TVC increased in both packaging methods, from 3.82 to 5.33 log CFU/g in the AP group and to 5.11 log CFU/g in the VP group. At each sampling day (0, 2, 5, 7, 9, 12, and 14) the TVCs were significantly higher (*p* < 0.05) compared with the previous sampling day. According to the recommendation of the ICMSF [97], the microbiological acceptability criterion for fresh fish is set at 6–7 log CFU/g, which confirms that the examined fish balls remained within the microbiological quality limit throughout the tested storage period. PBC increased during storage, reaching 4.12 log CFU/g in air-packed samples and 3.85 log CFU/g in vacuum-packed samples, compared with an initial value of 2.35 log CFU/g. Significant differences (*p* < 0.05) were also encountered between each sampling day. SPBC also showed an increase during the storage period from an initial value of 2.40 to 3.57 log CFU/g in fish balls packed in air and to 3.40 log CFU/g in products packed in a vacuum at 14 d. At each sampling day, the counts were significantly higher (*p* < 0.05) than those recorded at the previous sampling day. LABCs were increased during storage in samples packed with both methods, from 2.11 to 2.89 log CFU/g in the AP group and to 3.16 log CFU/g in the VP group, respectively. In air-packed samples, the growth of LABC was inhibited between 7 d and 12 d. This stagnation coincided with a temporary increase in pH observed in the AP group during the same period, suggesting that the reduced LABC activity may have allowed spoilage-associated bacteria to dominate and produce alkaline metabolites [98].

In contrast, LABCs in vacuum-packed samples continued to increase, which may have contributed to the more stable pH values recorded in this group. Inhibiting the growth of mesophilic and psychrophilic aerobic bacteria (TVC, PBC, SPBC) and lactic acid bacteria (LABC) is crucial not only for keeping the microbiological standard but also for maintaining the sensory qualities of the product, such as texture, odor, overall acceptability, and the TBARS stability and pH value [14]. Sahagún-Aguilar et al. [99] showed that proteolytic LAB species present on the skin and the gastrointestinal tract of common carp display high proteolytic activity, influenced by the ecosystem where they develop. The increased presence of these bacteria in vacuum-packed samples reflects their natural adaptation, which supports product stability and delays spoilage. Özpolat et al. [91] revealed that vacuum packaging can effectively inhibit the growth of the bacteria responsible for the spoilage of fish balls. According to their findings, the spoilage threshold (>7 log CFU/g) was reached solely at 14 d in samples packed in air, whereas samples packed in a vacuum reached this level at 35 d. In contrast, in the present study, the TVC remained below 6 log CFU/g even at 14 d. TSCs increased during refrigerated storage, from 2.08 log CFU/g to 3.26 log CFU/g in air-packed samples and to 3.01 log CFU/g in vacuum-packed samples. At each sampling day (0, 2, 5, 7, 9, 12, and 14), the TSC values were significantly higher (*p* < 0.05) compared with the previous sampling day. The presence and growth of TSC can negatively impact the safety and quality of the product by contributing to proteolysis and lipolysis. Noordin et al. [14] reported earlier that staphylococcal counts in cooked fish balls stayed below 3 log CFU/g during refrigerated storage and increased more slowly under vacuum packaging. The products tested negative for *Enterobacteriaceae* spp. (EBC), *E. coli* (EC), *Listeria* spp., and *Salmonella* spp. This result indicates a good hygienic quality and confirms the safety of the product.

## 3. Materials and Methods

### 3.1. Sample Collection and Fish Ball Preparation

The research material consisted of skin-on filets from freshly harvested carp (*C. carpio*, aged 3 years or older). The carp filets were fabricated in a certified fish farm located in Lublin Province (Poland) twice (2 replicates) in autumn 2022 at an interval of 21 days. In both cases, filets were collected from six carps, whose mean body weights were similar (*p* = 0.499), 1558 g and 1462 g, respectively. The farm conducts farming in a low-intensity method, where fish are maintained in earth ponds, and the feed is mainly natural food (benthos, zooplankton) with additional grain feeding (wheat, barley and rye). Filets were transported to the laboratory up to 2 h in a passive travel fridge at 2 °C (±2 °C) and processed immediately. The filets were microwaved at a power of 800–1200 W for 3–6 min [100]; then, the muscle tissue was separated from the skin. The muscle tissue was minced and mixed with salt (1%) and black pepper (0.2%). Semi-raw fish balls of uniform shape and size (average weight of 10 ± 0.1 g and diameter of 25 ± 1 mm) were prepared and randomly divided into two groups. The first group consisted of fish balls packed with contact with air (AP) using polypropylene plastic boxes and covered with food wrapping foil. The second group (VP) consisted of fish balls packed under a vacuum using a Multivac C200 tabletop chamber machine (MULTIVAC Sepp Haggenmüller SE & Co. KG, Wolfertschwenden, Germany) in 20/70 μm PA/PE side seal bags with oxygen permeability of <56 cm^3^/m^2^/24 h/bar at 50% RH and water vapor permeability of <3 g/m^2^/24 h at 85% RH (MULTIVAC, Bucharest, Romania). The samples were stored up to 14 days in refrigerated (+4 ± 1 °C) conditions and analyzed on the following days: 0, 2, 5, 7, 9, 12, and 14. A total of 28 sets of fish balls (two packaging methods × seven sampling days × two replicates) were prepared, and each set consisted of 16 individual semi-raw samples. The fish balls dedicated to texture and sensory analysis were deep-fried in refined cold-filtered virgin rapeseed oil (per 100 g: 3700 kJ/900 kcal, fat 100.0 g, saturated fatty acids 7.5 g, monounsaturated fatty acids 65.5 g, polyunsaturated fatty acids 26.5 g, omega 3 fatty acids 8.0 g, vit. E 25 mg, vit. K 75 µg) immediately before analyses. Samples for the remaining analyses were used in the semi-raw form. The most important stages of preparation, storage and processing of fish balls are shown in Figure S5.

### 3.2. Determination of Proximate Composition and Fatty Acids Profile

The proximate composition and the caloric value of raw carp meat and fish balls (before and after frying) were determined according to the methodology provided earlier by Kaliniak-Dziura et al. [101]. Briefly, moisture content was determined through drying (at 103 °C) according to PN-ISO 1442:2000 [102], total ash through incineration (at 550 °C) according to PN-ISO 936:2000 [103], total protein content (N × 6.25) through the Kjeldahl method according to PN-A-04018/Az3:2002 [104], and fat content through the Soxhlet method (with n-hexane as solvent) in accordance with PN-ISO 1444:2000 [105]. The samples were analyzed in duplicate. The caloric value of 100 g was calculated using the following physical energy equivalents: for 1 g protein, 23.64 kJ; and for 1 g fat, 39.54 kJ. Following fat extraction [106], the proportions of fatty acids were determined according to the methodology provided earlier by Skałecki et al. [68]. The chromatogram of identified fatty acid methyl esters in carp fish balls is presented in the Figure S6.

### 3.3. Measurement of pH and Water Activity

The pH was measured using a portable pH-meter with automatic buffer solution detection and automatic temperature compensation (CP-401, Elmetron, Zabrze, Poland) and a penetrating glass electrode (ERH-12-6, Hydromet, Gliwice, Poland) calibrated at pH = 4.00 and pH = 7.00 with high-accuracy pH buffer solutions (± 0.02 at +20 °C, Elmetron, Zabrze, Poland). The analysis was performed in duplicate, directly in the product. Water activity was measured using a Rotronic HygroLab C1 analyzer (Bassersdorf, Switzerland). The AWQ mode with stabilization set to 15 min was applied after conditioning the samples at room temperature (+20 ± 1 °C). For each sample, two replicates were recorded.

### 3.4. Oil Absorption

The oil absorption (%) was calculated according to the following equation:

OA = (weight of sample after frying − weight of sample before frying) × 100/weight of sample before frying(1)

The fish balls were weighed before and after deep-frying using a digital balance. This was carried out in three replicates, and the average weight was calculated.

### 3.5. Color Measurement

Fish ball color was measured using the Konica Minolta CM-600d (Konica Minolta Sensing, Inc., Osaka, Japan) portable spectrophotometer with a pulsed xenon lamp and 8 mm aperture size. The results (illuminant D65, 10° Standard Observer) are presented under the CIE L*a*b* color space [107], including the following spectral values: L*, (lightness), a* (redness), b* (yellowness), and total color difference (∆E). The color of the fish balls was measured on the external surface (before and after thermal treatment) and on the cross-section (after thermal treatment). At least two readings on each surface of the sample were performed.

### 3.6. Texture Analysis

Texture analysis was performed on deep-fried fish balls (n = 5) using the Zwick/Roell ProLine BDO125 FB0.5TS (Zwick GmbH and Co., Ulm, Germany) equipped with a Warner–Bratzler V-shaped shear blade and a crosshead with a 500 N load cell, with the speed set at 100 mm/min. The maximal shear force (N) and shear energy (mJ) were recorded using device-specific testXpert II software.

### 3.7. Determination of Acid Value and Peroxide Value

Following fat extraction [106], the acid value was determined in accordance with PN-EN ISO 660:2021-03 [108] through the titration method and expressed in mg KOH/g fat, and the peroxide value was determined according to PN-EN ISO 3976:2014-11 [109]. The absorbance was measured at 500 nm using the Varian Cary 300 Bio spectrophotometer (Varian Australia PTY Ltd., Mulgrave, Australia), and the results were expressed in mEq O_2_/kg fat.

### 3.8. Determination of TBARS Index

The 2-thiobarbituric acid reactive substances (TBARS) were determined according to Witte et al. [110]. The absorbance was measured at 530 nm using the Varian Cary 300 Bio spectrophotometer (Varian Australia PTY Ltd., Mulgrave, Australia) and expressed in mg of malondialdehyde (MDA) per kg of sample.

### 3.9. Sensory Evaluation

Sensory analysis of fish balls was performed on fresh, deep-fried, warm samples (+50 °C). Six non-smokers (3 women and 3 men in an age range of 25 to 50 years) selected from the staff of the Department of Quality Assessment and Processing of Animal Products, University of Life Sciences in Lublin were used as a panel. Before analysis, the panel took part in a preparatory session concerning sensory attributes to allow the panelists to discuss and clarify each attribute to be evaluated. Samples were coded with three-digit numbers and served. All testing was carried out under controlled conditions in room temperature (approx. 20 °C), free of noise and odor, under fluorescent lightning. Water and bread were provided between samples to cleanse the palate. The sensory analysis was carried out using the scaling method according to ISO 4121:2003 [111]. A 10-point unstructured linear graphical scale (10 cm) with a specific edge point was used to assess the following attributes: fish smell (undetectable; very intense), fish taste (undetectable; very intense), juiciness (very dry; very juicy), tenderness (very tough; very tender), and overall quality (highly undesirable; extremely desirable). Each panelist participated in seven sessions (subsequent sampling days) per replication and during each session evaluated three sub-samples from each group in randomized order. Sensory cutoff thresholds have been defined for the overall quality. For this indicator, on a scale of 0–10 points, a value of 5 is recommended. If the mean of the scores was below this value, the batch of fish balls assessed on a given day in a given packaging system was disqualified (“not acceptable”). Definitions and scales for each attribute used for the sensory analysis are presented in Table 6.

### 3.10. Microbiological Analysis

For microbiological examination, six fish balls (10 g each) were assessed from each packaging method (AP, n = 6; VP, n = 6) on days 0, 2, 5, 7, 9, 12, and 14. Additionally, 25 g samples were collected for the detection of *Salmonella* and *Listeria*. A 10 g sample was transferred into sterile stomacher bags containing 90 mL of saline peptone water (BioMaxima, Lublin, Poland) and homogenized for 2 min at 230 rpm, following PN-EN ISO 6887-2:2017-05 [112] and PN-EN ISO 7218:2008 [113] standards. Serial decimal dilutions were prepared from the initial 10^−1^ suspension and inoculated onto appropriate selective media. Total viable counts (TVCs) and psychrotrophic bacterial counts (PBCs) were determined on Plate Count Agar (BioMaxima, Poland) after incubation at 30 °C for 48 h and at 0–4 °C for 14 days, respectively [114,115]. *Enterobacteriaceae* (EBC) were quantified on Violet Red Bile Glucose Agar (BioMaxima, Poland) after incubation at 37 °C for 24 h [116]. *Escherichia coli* (EC) was enumerated on Tryptone Bile X-glucuronide Agar (BioMaxima, Poland) with incubation at 44 °C for 24 h [117]. Total staphylococcal counts (TSC) were assessed on RPF Agar (BioMaxima, Poland) after incubation at 37 °C for 24–48 h [118]. Sulfite-producing bacteria (SPBC) were recovered on Iron Agar following incubation at 35 °C for 48 h [119]. Lactic acid bacteria counts (LABCs) were grown on de Man, Rogosa, and Sharpe (MRS; BioMaxima, Poland) agar and incubated at 30 °C for 48 h [120]. All bacterial populations were determined as the log of colony-forming units (log CFU g^−1^). Isolation and determination of *Salmonella* spp. were performed according to PN-ISO 6579-1:2017-04 [121]. In order to determine the presence of *Listeria* spp., an examination was performed, guided by PN-EN ISO 11290-1:2017-07 [122].

### 3.11. Statistical Analysis

The statistical analysis was performed using Statistica software (ver. 13.1, TIBCO Software Inc., Palo Alto, CA, USA). Data were subjected to a two-way ANOVA with packaging method (AP vs. VP), storage day (2, 5, 7, 9, 12 and 14), and their interactions as fixed factors, while the replicates were set as a random factor. The means of variables among different groups were compared using Tukey’s (HSD) test, and the level of significance was set at *p* < 0.05. Main effects and interactions of tested factors on the properties of fish balls are presented in Table 7. In addition, the one-way ANOVA and Dunnett’s test were applied for multiple comparisons with the control value (0 d) within the packaging method. The microbial counts were log-transformed and then analyzed using one-way ANOVA, followed by Tukey’s (HSD) post hoc test when significant differences (*p* < 0.05) were detected.

## 4. Conclusions

The obtained results showed that vacuum packaging may effectively slow down the increase in PV and TBARS. Moreover, VP could maintain a more favorable OA, pH and most color indices of fish balls during refrigerated storage. According to the results of the overall sensory assessment, carp fish balls kept at +4 °C were acceptable for up to 9 days when stored in air, and 12 days packed under a vacuum. Vacuum packaging effectively suppressed the growth of aerobic bacteria (TVC, PBC, SPB, TSC) while promoting LAB development, which contributed to pH stabilization, slower oxidative changes (TBARS, AV, PV), prolonged sensory acceptability, and an extended shelf life. For a better understanding of microbial community dynamics and their impact on product stability, we will consider the use of high-throughput 16S rRNA sequencing in future research. In conclusion, the application of vacuum packaging can improve the quality of carp fish balls and prolong their refrigerated shelf life. Moreover, the development of convenient food items from commonly farmed fish species may represent an effective strategy to enhance consumer approval, promote the better use of cyprinid species, and contribute to more sustainable aquaculture practices.

## Figures and Tables

**Table 1:** Proximate composition (%), energy value (kJ/100 g) and partial sums of fatty acids (% of total fatty acids) for raw carp (*Cyprinus carpio*) meat, semi-raw and deep-fried fish balls (mean ± standard deviation).

Ingredient	Raw Carp Meat	Carp Fish Balls	
		Semi-Raw	Deep-Fried
Moisture	74.7 ^c^ ± 0.2	69.6 ^b^ ± 0.5	57.0 ^a^ ± 0.6
Protein	18.4 ^a^ ± 0.7	21.5 ^b^ ± 0.7	25.8 ^c^ ± 1.0
Lipids	5.7 ^a^ ± 0.6	6.8 ^a^ ± 0.1	14.5 ^b^ ± 0.7
Ash	1.2 ^a^ ± 0.1	2.2 ^b^ ± 0.0	2.8 ^c^ ± 0.4
Energy value	522 ^a^ ± 10	615 ^b^ ± 7	973 ^c^ ± 17
Fatty acids			
Saturated (SFA)	27.0 ^b^ ± 1.7	29.1 ^b^ ± 0.1	18.3 ^a^ ± 1.4
Monounsaturated (MUFA)	62.1 ^b^ ± 1.8	56.3 ^a^ ± 0.2	60.2 ^b^ ± 1.5
Polyunsaturated (PUFA)	10.4 ^a^ ± 0.8	12.8 ^b^ ± 0.5	20.1 ^c^ ± 0.1

^a, b, c^—Means indicated by different letters within a row are significantly different (*p* < 0.05).

**Table 2:** The physicochemical and texture changes in carp (*Cyprinus carpio*) fish balls stored at 4 °C under air (AP) and vacuum (VP) (mean ± standard deviation).

Parameter	Packaging Method	Storage Day						
		0	2	5	7	9	12	14
pH	AP	6.81 ± 0.02	6.74 ± 0.05 ^b^^	6.78 ± 0.04 ^cd^	6.90 ± 0.02 ^f^^	6.77 ± 0.01 ^bcd^	6.76 ± 0.02 ^bc^	6.68 ± 0.03 ^a^^
	VP		6.81 ± 0.02 ^cde^	6.79 ± 0.03 ^cd^	6.84 ± 0.02 ^e^	6.81 ± 0.01 ^cde^	6.84 ± 0.03 ^e^	6.81 ± 0.02 ^de^
OA [%]	AP	27.18 ± 1.24	30.30 ± 2.31 ^^^	31.42 ± 0.60 ^^^	32.23 ± 1.63 ^^^	34.80 ± 3.24 ^^^	35.59 ± 1.05 ^^^	35.40 ± 1.56 ^^^
	VP		30.50 ± 0.50 ^#^	29.71 ± 0.81 ^#^	30.47 ± 1.43 ^#^	32.10 ± 1.87 ^#^	32.68 ± 0.86 ^#^	35.61 ± 0.32 ^#^
WBSF [N]	AP	45.3 ± 6.6	66.27 ± 14.06 ^b^	56.97 ± 11.30 ^ab^	49.07 ± 5.65 ^ab^	50.80 ± 11.35 ^ab^	49.13 ± 5.09 ^ab^	41.33 ± 2.86 ^a^
	VP		45.65 ± 2.47 ^ab^	43.40 ± 0.99 ^ab^	37.10 ± 0.14 ^a^	54.75 ± 0.07 ^ab^	54.35 ± 2.05 ^ab^	50.50 ± 0.28 ^ab^
Shear energy [mJ]	AP	495.61 ± 26.97	821.95 ± 357.49	635.65 ± 168.05	439.28 ± 109.63	658.26 ± 48.22	804.12 ± 80.58	555.07 ± 115.16
	VP		497.04 ± 144.09	488.23 ± 305.99	586.40 ± 25.09	760.20 ± 49.56	587.61 ± 150.27	688.02 ± 57.83

OA—oil absorption; WBSF—Warner–Bratzler shear force. ^a, b, c, d, e, f^—For each parameter, means with different superscripts are significantly (*p* < 0.05) different (day 0 was included in this analysis); ^^^—means with this symbol under the air packaging method are significantly different from day 0 (*p* < 0.05); ^#^—means with this symbol under the vacuum pressure method are significantly different from day 0 (*p* < 0.05).

**Table 3:** The CIE L*a*b* color of semi-raw and deep-fried carp (*Cyprinus carpio*) fish balls stored at 4 °C under air (AP) and vacuum (VP) (mean ± standard deviation).

CIE Coordinate	Packaging	Storage Day						
		0	2	5	7	9	12	14
Semi-raw fish balls								
L*	AP	68.72 ± 0.67	69.57 ± 0.64 ^abcd^^	70.54 ± 0.60 ^ef^^	69.53 ± 1.08 ^abcd^^	69.82 ± 0.63 ^bcde^^	71.13 ± 0.26 ^f^^	69.85 ± 0.81 ^cde^^
	VP		69.08 ± 0.56 ^abcd^	69.09 ± 0.70 ^abc^	69.24 ± 0.56 ^abcd^	68.69 ± 0.31 ^a^	68.94 ± 0.86 ^ab^	69.83 ± 0.59 ^de#^
a*	AP	0.72 ± 0.33	0.40 ± 0.15 ^c^^	0.26 ± 0.15 ^bc^^	0.18 ± 0.19 ^b^^	0.13 ± 0.16 ^ab^^	−0.01 ± 0.10 ^a^^	−0.03 ± 0.10 ^a^^
	VP		0.66 ± 0.16 ^d^	0.88 ± 0.14 ^e^	0.90 ± 0.05 ^e^	0.88 ± 0.06 ^e^	0.83 ± 0.20 ^e^	0.85 ± 0.09 ^de^
b*	AP	14.26 ± 0.53	15.60 ± 0.35 ^^^	15.77 ± 0.34 ^^^	15.58 ± 0.76 ^^^	15.61 ± 0.27 ^^^	15.81 ± 0.36 ^^^	15.57 ± 0.36 ^^^
	VP		15.79 ± 0.57 ^#^	15.81 ± 0.28 ^#^	15.89 ± 0.33 ^#^	16.00 ± 0.47 ^#^	15.53 ± 0.53 ^#^	15.91 ± 0.21 ^#^
∆E	AP	n.a.	1.03 ± 0.55	0.94 ± 0.69	1.35 ± 0.96	0.81 ± 0.43	0.70 ± 0.39	0.81 ± 0.47
	VP		0.99 ± 0.45	0.87 ± 0.47	0.81 ± 0.53	0.85 ± 0.53	1.44 ± 1.08	0.71 ± 0.53
		Deep-fried fish balls (external surface)						
L*	AP	56.41 ± 2.05	56.68 ± 1.90	54.85 ± 1.58	54.14 ± 2.00 ^^^	53.34 ± 1.97 ^^^	53.34 ± 1.65 ^^^	50.89 ± 2.67 ^^^
	VP		55.66 ± 2.44	54.99 ± 2.07	53.82 ± 1.27 ^#^	53.44 ± 1.86 ^#^	53.12 ± 1.60 ^#^	51.81 ± 1.41 ^#^
a*	AP	5.54 ± 0.44	6.62 ± 0.57 ^a^^	9.38 ± 0.44 ^c^^	11.77 ± 0.73 ^e^^	12.80 ± 0.94 ^fg^^	13.11 ± 0.76 ^g^^	14.83 ± 0.67 ^h^^
	VP		6.65 ± 0.85 ^a#^	7.07 ± 1.34 ^ab#^	7.68 ± 1.45 ^b#^	9.84 ± 0.87 ^c#^	10.82 ± 0.89 ^d#^	11.93 ± 0.79 ^ef#^
b*	AP	25.95 ± 2.12	28.22 ± 0.93 ^efg^^	26.04 ± 0.69 ^cde^	26.36 ± 1.36 ^cde^	26.56 ± 3.96 ^cde^	25.48 ± 1.57 ^bcd^	31.20 ± 2.15 ^h^^
	VP		30.08 ± 1.50 ^gh#^	27.19 ± 3.05 ^def^	23.63 ± 1.25 ^ab#^	23.46 ± 1.20 ^a#^	24.82 ± 1.15 ^abc^	28.68 ± 1.28 ^fg#^
∆E	AP	n.a.	1.88 ± 1.16 ^a^	1.85 ± 1.44 ^ab^	3.44 ± 2.31 ^ab^	3.77 ± 3.65 ^ab^	2.95 ± 1.31 ^ab^	3.90 ± 3.14 ^ab^
	VP		3.81 ± 2.08 ^ab^	4.43 ± 2.19 ^b^	2.76 ± 1.45 ^ab^	3.35 ± 1.63 ^ab^	2.85 ± 1.55 ^ab^	2.47 ± 1.76 ^ab^
		Deep-fried fish balls (internal surface)						
L*	AP	65.37 ± 1.64	64.91 ± 2.88	64.78 ± 2.73	65.07 ± 2.94	64.78 ± 3.30	64.98 ± 2.68	63.58 ± 2.31
	VP		64.73 ± 1.96	66.40 ± 2.30	66.26 ± 3.52	66.46 ± 2.51	64.53 ± 3.13	60.60 ± 4.14 ^#^
a*	AP	1.11 ± 0.21	0.84 ± 0.15	1.22 ± 0.16	1.39 ± 0.24	1.15 ± 0.27	1.04 ± 0.42	2.38 ± 1.09 ^^^
	VP		1.17 ± 0.30	1.64 ± 1.07	1.70 ± 1.19	1.31 ± 0.28	1.22 ± 0.56	1.84 ± 0.88
b*	AP	15.97 ± 0.79	15.45 ± 0.89 ^a^	17.51 ± 0.90 ^ab^	15.89 ± 0.95 ^a^	17.03 ± 0.80 ^ab^	16.48 ± 1.42 ^ab^	22.48 ± 2.59 ^c^^
	VP		16.45 ± 0.88 ^ab^	17.42 ± 1.92 ^ab^	16.68 ± 1.67 ^ab^	16.89 ± 1.03 ^ab^	16.03 ± 2.08 ^a^	18.78 ± 2.36 ^b#^
∆E	AP	n.a.	3.11 ± 2.54	3.07 ± 2.61	4.01 ± 2.34	3.11 ± 1.74	3.51 ± 1.71	5.69 ± 2.32
	VP		3.28 ± 1.77	3.74 ± 2.08	4.21 ± 2.84	2.96 ± 1.75	4.52 ± 2.40	4.78 ± 2.15

L*—lightness; a*—redness; b*—yellowness; ∆E—total color difference; semi-raw fish balls—samples after microwaved pre-treatment and salt and pepper addition. ^a, b, c, d, e, f, g, h^—For each parameter, means with different superscripts are significantly (*p* < 0.05) different (day 0 was not included in this analysis); ^^^—means with this symbol under the air packaging method are significantly different from day 0 (*p* < 0.05); ^#^—means with this symbol under the vacuum pressure method are significantly different from day 0 (*p* < 0.05); n.a.—not applicable.

**Table 4:** The sensory properties (rated on a 10-point scale) of carp (*Cyprinus carpio*) fish balls stored at 4 °C under air (AP) and vacuum (VP) (mean ± standard deviation).

Attribute	Packaging	Storage Day						
		0	2	5	7	9	12	14
Fish smell	AP	4.3 ± 1.9	5.2 ± 2.8	5.4 ± 2.2	6.0 ± 3.0	6.7 ± 2.9	7.0 ± 3.4	7.3 ± 2.8
	VP		5.0 ± 1.8	5.3 ± 3.5	5.9 ± 2.4	5.9 ± 2.3	6.5 ± 1.8	6.4 ± 2.9
Fish taste	AP	5.7 ± 1.6	5.7 ± 1.9	6.1 ± 1.1	6.8 ± 2.3	6.9 ± 2.4	7.7 ± 2.4	7.4 ± 3.7
	VP		5.5 ± 2.6	6.3 ± 2.0	6.7 ± 1.5	6.1 ± 2.2	6.2 ± 1.8	7.3 ± 0.7
Juiciness	AP	4.6 ± 2.8	5.0 ± 2.4	5.2 ± 2.5	5.2 ± 2.6	6.4 ± 1.5	5.1 ± 1.5	4.2 ± 0.7
	VP		6.1 ± 1.8	6.1 ± 1.8	6.7 ± 1.2	7.1 ± 1.1	5.8 ± 1.3	5.2 ± 0.3
Tenderness	AP	5.6 ± 1.8	5.6 ± 1.4	6.8 ± 0.5	6.9 ± 0.1	7.1 ± 1.4	6.8 ± 1.7	4.2 ± 0.7
	VP		6.0 ± 1.9	7.0 ± 0.9	7.2 ± 1.2	7.5 ± 1.6	7.1 ± 1.8	5.7 ± 0.9
Overall quality	AP	7.7 ± 1.5	7.1 ± 0.7	7.3 ± 0.5	6.6 ± 1.1	6.8 ± 2.9	4.9 ± 2.0 ^^^	3.1 ± 2.3 ^^^
	VP		7.6 ± 1.1	7.6 ± 1.1	7.3 ± 0.9	7.9 ± 0.5	6.2 ± 1.1	4.9 ± 2.0 ^#^

^^^—Means with this symbol under the air packaging method are significantly different from day 0 (*p* < 0.05); ^#^—means with this symbol under the vacuum pressure method are significantly different from day 0 (*p* < 0.05).

**Table 5:** The microbial status of carp (*Cyprinus carpio*) fish balls stored at 4 °C under air (AP) and vacuum (VP) (mean ± standard deviation).

Microbial Group	Packaging	Storage Day						
		0	2	5	7	9	12	14
TVC	AP	3.82 ^a^ ± 0.21	4.27 ^b^ ± 0.10	4.61 ^c^ ± 0.11	4.79 ^d^ ± 0.20	5.02 ^e^ ± 0.17	5.19 ^f^ ± 0.07	5.33 ^g^ ± 0.18
	VP		3.95 ^b^* ± 0.14	4.45 ^c^* ± 0.07	4.58 ^d^* ± 0.10	4.66 ^e^* ± 0.05	4.89 ^f^*± 0.05	5.11 ^g^* ± 0.05
PBC	AP	2.35 ^a^ ± 0.21	2.50 ^b^ ± 0.21	3.19 ^c^ ± 0.15	3.51 ^d^ ± 0.10	3.71 ^e^ ± 0.09	3.85 ^f^ ± 0.07	4.12 ^g^ ± 0.05
	VP		2.39 ^b^* ± 0.20	2.98 ^c^*± 0.17	3.41 ^d^* ± 0.10	3.59 ^e^* ± 0.10	3.67 ^f^*± 0.05	3.85 ^g^* ± 0.07
TSC	AP	2.08 ^a^ ± 0.31	2.20 ^b^ ± 0.19	2.62 ^c^ ± 0.22	2.85 ^d^ ± 0.15	2.96 ^e^ ± 0.07	3.13 ^f^ ± 0.07	3.26 ^g^ ± 0.05
	VP		2.22 ^b^ ± 0.15	2.50 ^c^* ± 0.22	2.48 ^c^* ± 0.22	2.76 ^d^* ± 0.15	2.84 ^e^* ± 0.12	3.01 ^f^* ± 0.10
SPBC	AP	2.40 ^a^ ± 0.21	2.42 ^b^ ± 0.17	2.78 ^c^ ± 0.12	3.05 ^d^ ± 0.10	3.17 ^e^ ± 0.15	3.46 ^f^ ± 0.05	3.57 ^g^ ± 0.07
	VP		2.42 ^b^ ± 0.15	2.49 ^c^* ± 0.19	2.77 ^d^* ± 0.14	3.06 ^e^* ± 0.12	3.30 ^f^* ± 0.05	3.40 ^g^* ± 0.07
LABC	AP	2.11 ^a^ ± 0.18	2.32 ^b^ ± 0.10	2.37 ^c^ ± 0.17	2.54 ^d^ ± 0,05	2.56 ^d^ ± 0.17	2.56 ^d^ ± 0.10	2.89 ^e^ ± 0.12
	VP		2.49 ^b^* ± 0.12	2.67 ^c^* ± 0.17	2.85 ^d^* ± 0.05	2.93 ^e^* ± 0.12	3.07 ^f^* ± 0.07	3.16 ^g^* ± 0.10

TVC—total viable count; PBC—psychrotrophic bacterial couns; TSC—total staphylococcal count; SPBC—sulfite-producing bacteria; LABC—lactic acid bacteria; EBC—*Enterobacteriaceae* spp.; EC—*Escherichia coli*; *Enterobacteriaceae*, *Escherichia coli* < 100 cfu/g—the result below the detection limit of the method; *Listeria* spp., *Salmonella* spp.—not detected in tested samples. a, b, c, d, e, f, g—Means indicated by different letters are significantly different horizontally within the storage days (*p* < 0.05); *—marked means are significantly different between AP and VP for tested day in a particular group of bacteria.

**Table 6:** Definition and scale of each attribute used for the sensory analysis.

Sensory Attributes	Characterization	Scale
Fish smell	Aroma associated with fish in deep-fried product	0 = undetectable 10 = very intense
Fish taste	Flavor associated with fish meat prior to swallowing	0 = undetectable 10 = very intense
Juiciness	The level of juiciness perceived after the first 5 chews using the molar teeth	0 = very dry 10 = very juicy
Tenderness	The impression of tenderness perceived after the first 5 chews using the molar teeth	0 = very tough 10 = very tender
Overall quality	A summary impression resulting from the perception of all product attributes	0 = highly undesirable 10 = extremely desirable

**Table 7:** Main effects and interaction of tested factors on properties of fish balls.

Parameters	Packaging Method	Storage Day	Interaction
pH	+	+	+
a_w_	−	−	−
OA [%]	+	+	−
WBSF [N]	−	−	+
Shear Energy [mJ]	−	−	−
AV	−	+	+
PV	+	+	+
TBARS	+	+	+
Color of semi-raw fish balls			
L*	+	+	+
a*	+	+	+
b*	+	−	−
Color of deep-fried fish balls (external surface)			
L*	−	+	−
a*	+	+	+
b*	+	+	+
Color of deep-fried fish balls (internal surface)			
L*	−	+	−
a*	−	+	−
b*	−	+	+
Sensory attributes			
Fish smell	−	−	−
Fish taste	−	−	−
Juiciness	+	−	−
Tenderness	−	+	−
Overall quality	+	+	−

OA—oil absorption; WBSF—Warner–Bratzler shear force; a_w_—water activity; L*—lightness; a*—redness; b*—yellowness; “+”—indicated the presence of a main effects or interaction of tested factors, “−”—indicated the absence of a main effects or interaction of tested factors.

## Data Availability

The original contributions presented in this study are included in the article/Supplementary Material. Further inquiries can be directed to the corresponding authors.

## Supplementary Materials

The following supporting information can be downloaded at: https://www.mdpi.com/article/10.3390/molecules31040746/s1 Figure S1. The physicochemical and texture changes of carp (*Cyprinus carpio*) fish balls stored at 4 °C under air (AP) and vacuum (VP) (mean ± standard deviation); Figure S2. The CIE L*a*b* colour of semi-raw carp (*Cyprinus carpio*) fish balls stored at 4 °C under air (AP) and vacuum (VP); Figure S3. The CIE L*a*b* colour of external surface od deep-fried carp (*Cyprinus carpio*) fish balls stored at 4 °C under air (AP) and vacuum (VP); Figure S4. The CIE L*a*b* colour of internal surface of deep-fried carp (*Cyprinus carpio*) fish balls stored at 4 °C under air (AP) and vacuum (VP); Figure S5. Preparation of fish balls; Figure S6. Chromatogram of the separation of fatty acid methyl esters in carp fish balls.

## Abbreviations

The following abbreviations are used in this manuscript:

AP	Air packaging
VP	Vacuum packaging
a_w_	Water activity
OA	Oil absorption
WBSF	Warner–Bratzler shear force
L*	Lightness
a*	Redness
b*	Yellowness
∆E	Total color difference
PV	Peroxide value
TBARS	Thiobarbituric acid reactive substances
MDA	Malondialdehyde
AV	Acid value
TVC	Total viable count
SPBC	Sulfite-producing bacteria count
PBC	Psychrotrophic bacterial count
TSC	Total staphylococcal count
LABC	Lactic acid bacteria count
EBC	*Enterobacteriaceae* spp.
EC	*Escherichia coli*
ICMSF	International Commission on Microbiological Specifications for Foods
EFSA	European Food Safety Authority
CAC	Codex Alimentarius Commission
CIE	Commission International de l’Eclairage
SFA	Saturated fatty acid
MUFA	Monounsaturated fatty acid
PUFA	Polyunsaturated fatty acid
