# Supplementary material for: Effect of Packaging Method (Under Vacuum vs. Air) on Quality and Shelf Life of Carp (Cyprinus caprio) Fish Balls Stored at Fridge for 14 Days
Source: Molecules. 2026 Feb 22;31(4):746. doi: 10.3390/molecules31040746 (PMC12942997; doi:10.3390/molecules31040746)
Supplement: Supplementary file 1 [file molecules-31-00746-s001.zip › molecules-4086720-supplementary.pdf]

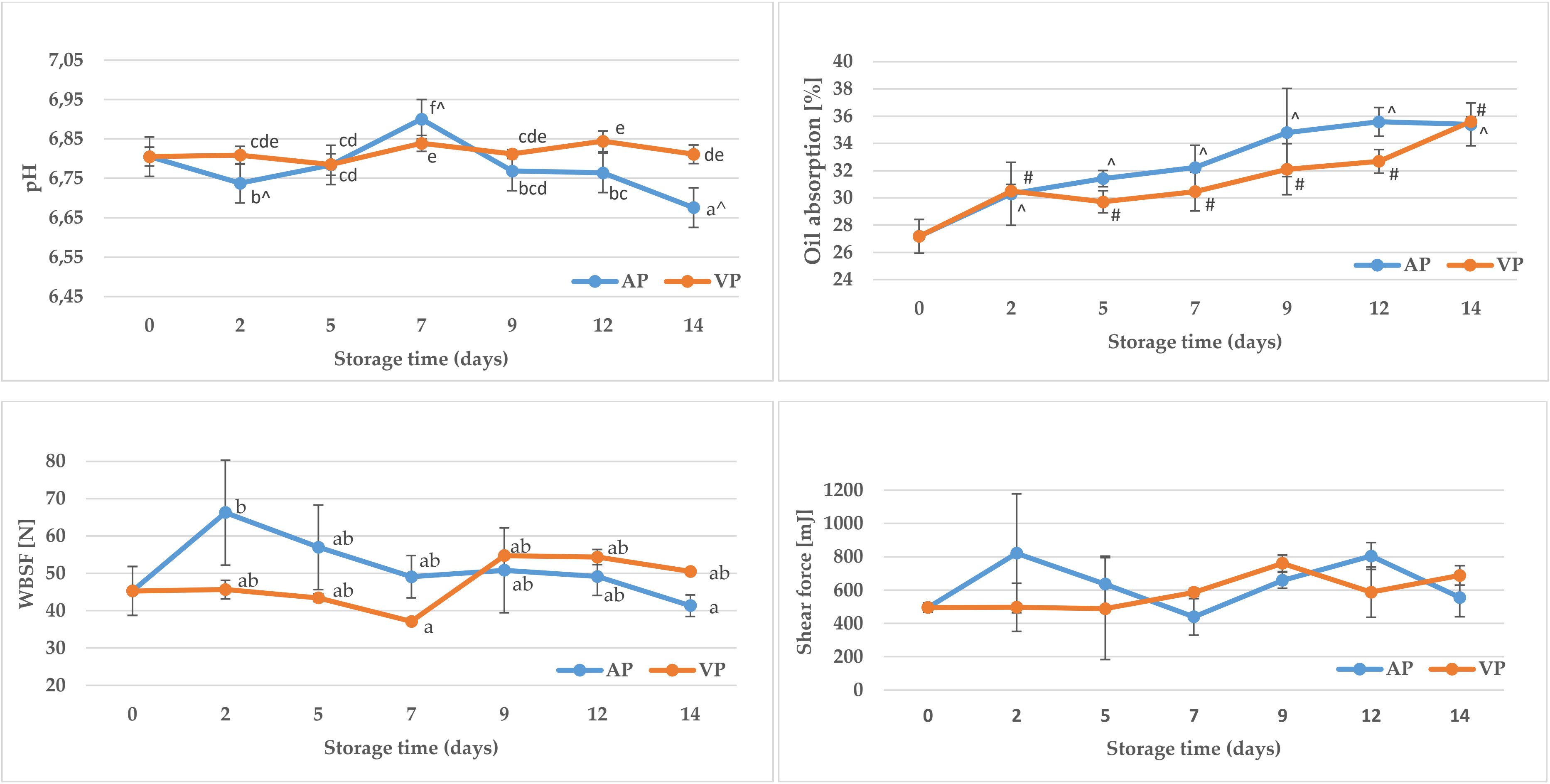

Figure S1. The physicochemical and texture changes of carp (*Cyprinus carpio*) fish balls stored at 4 °C under air (AP) and vacuum (VP) (mean ± standard deviation).

OA – oil absorption; WBSF – Warner-Bratzler shear force

a, b, c, d, e, f – For each parameter means with different superscripts are significantly ($p < 0.05$) different (day 0 was in included in this analysis)

^ – Means with the symbol under the air-packaging method are significantly different with the day 0 ($p < 0.05$)

# – Means with the symbol under the vacuum pressure method are significantly different with the day 0 ($p < 0.05$)

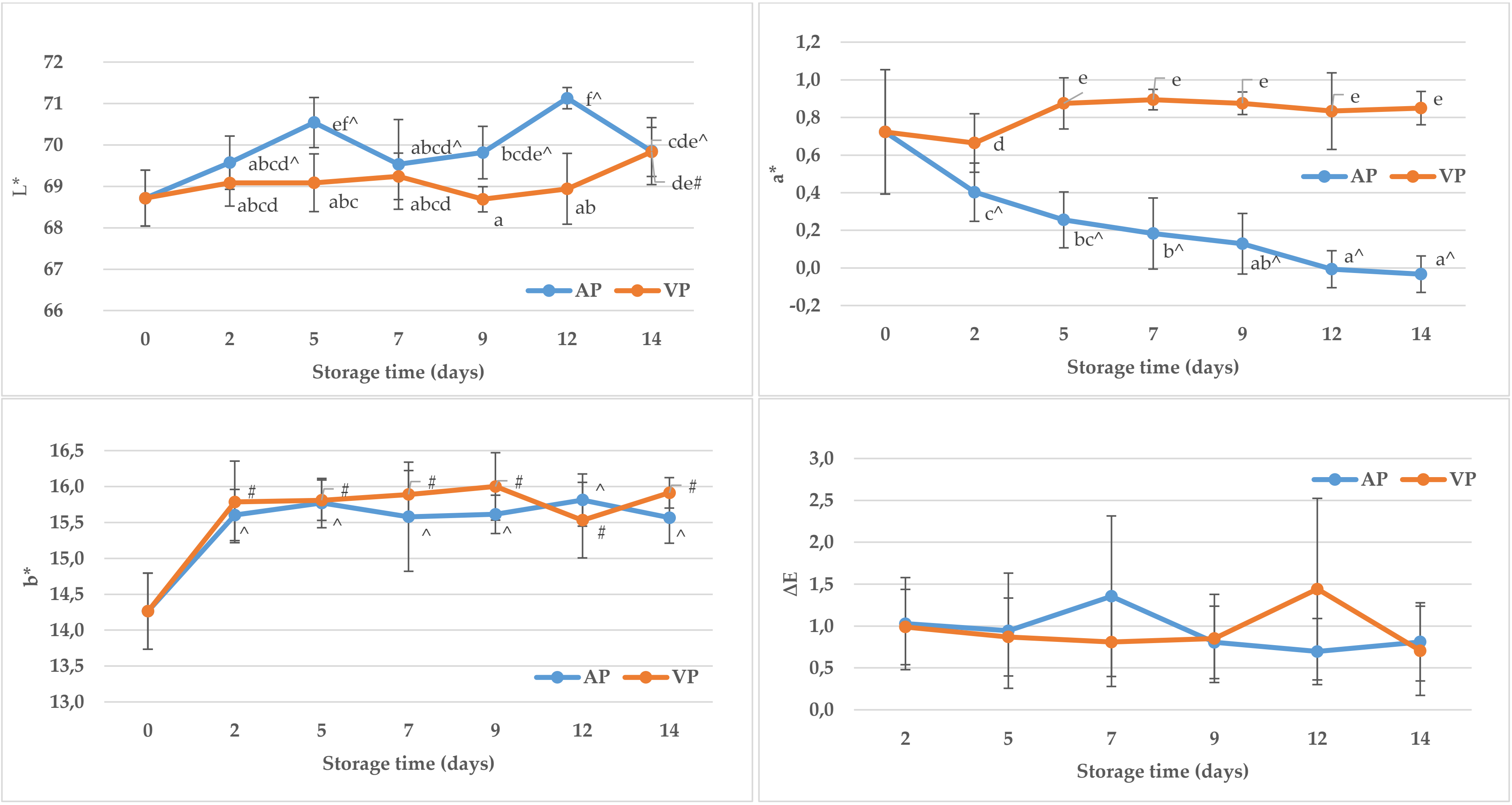

Figure S2. The CIE L*a*b* colour of semi-raw carp (*Cyprinus carpio*) fish balls stored at 4 °C under air (AP) and vacuum (VP).

L* – lightness; a* – redness; b* – yellowness; ΔE – total colour difference

a, b, c, d – Means with different superscripts are significantly different ($p < 0.05$)

^ – Means with the symbol under the air-packaging method are significantly different with the day 0 ($p < 0.05$)

# – Means with the symbol under the vacuum pressure method are significantly different with the day 0 ($p < 0.05$)

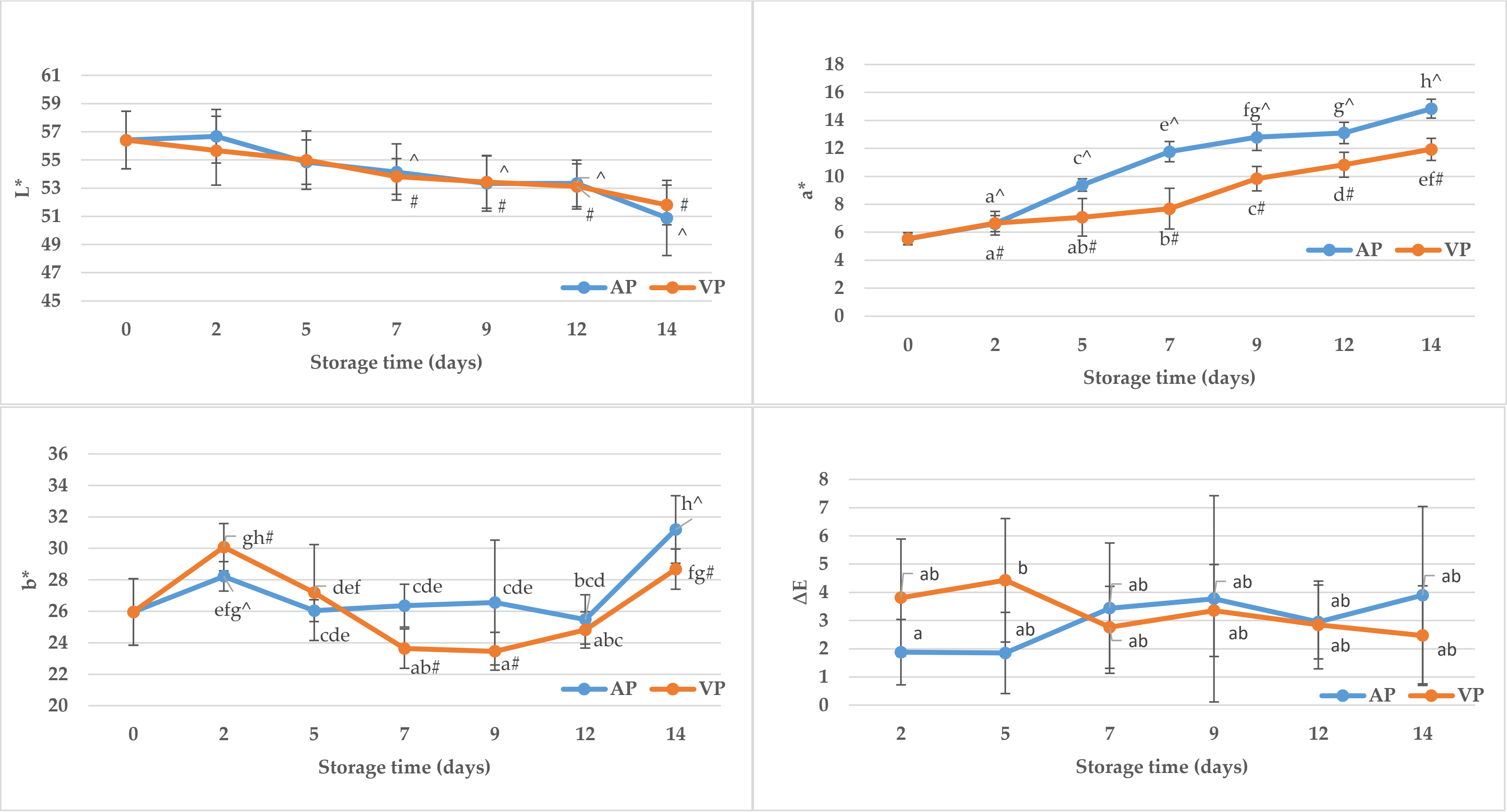

Figure S3. The CIE L*a*b* colour of external surface od deep-fried carp (*Cyprinus carpio*) fish balls stored at 4 °C under air (AP) and vacuum (VP).

L* – lightness; a* – redness; b* – yellowness; ΔE – total colour difference
a, b, c, d, e, f, g, h – Means with different superscripts are significantly different ($p < 0.05$)
^ – Means with the symbol under the air-packaging method are significantly different with the day 0 ($p < 0.05$)
# – Means with the symbol under the vacuum pressure method are significantly different with the day 0 ($p < 0.05$)

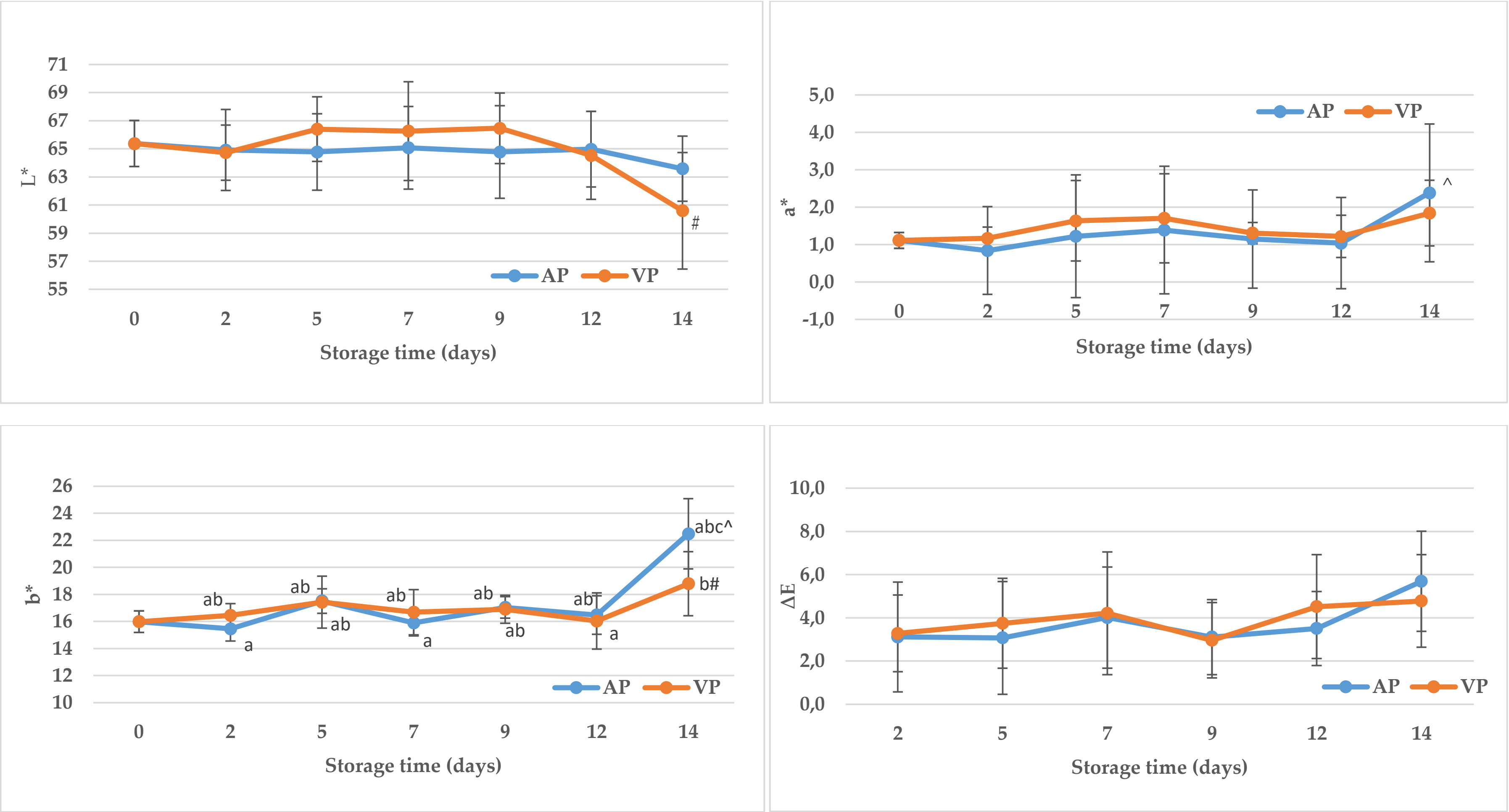

Figure S4. The CIE L*a*b* colour of internal surface of deep-fried carp (*Cyprinus carpio*) fish balls stored at 4 °C under air (AP) and vacuum (VP).

L* – lightness; a* – redness; b* – yellowness; ΔE – total colour difference
a, b, c, d – Means with different superscripts are significantly different ($p < 0.05$)
^ – Means with the symbol under the air-packaging method are significantly different with the day 0 ($p < 0.05$)
# – Means with the symbol under the vacuum pressure method are significantly different with the day 0 ($p < 0.05$)

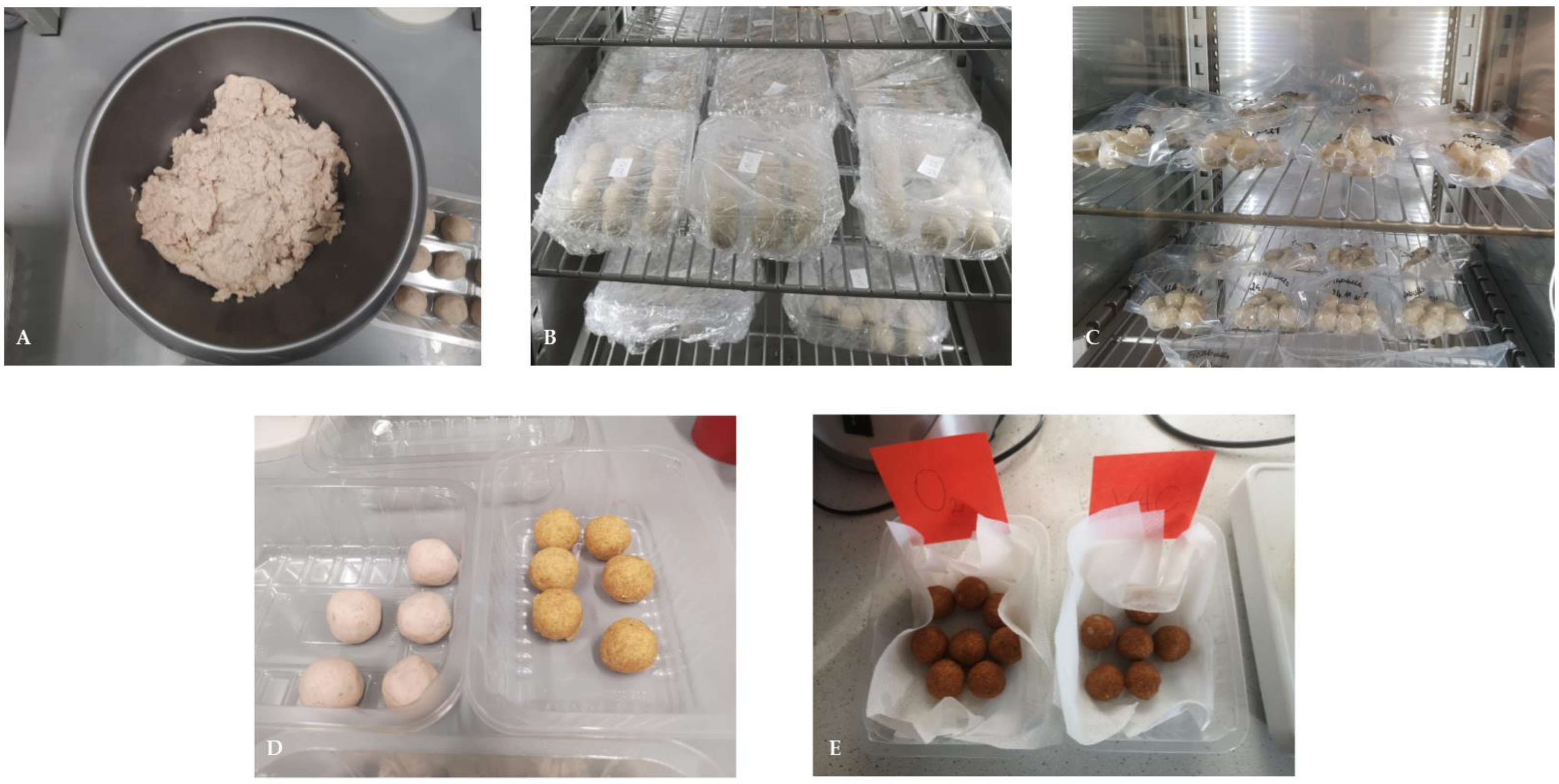

Figure S5. Preparation of fish balls. A—Fish mince; B— Air–packed fish balls during storage; C—Vacuum–packed fish balls during storage; D —Raw vs. fried samples; E—Air– and vacuum–packed samples after deep-frying

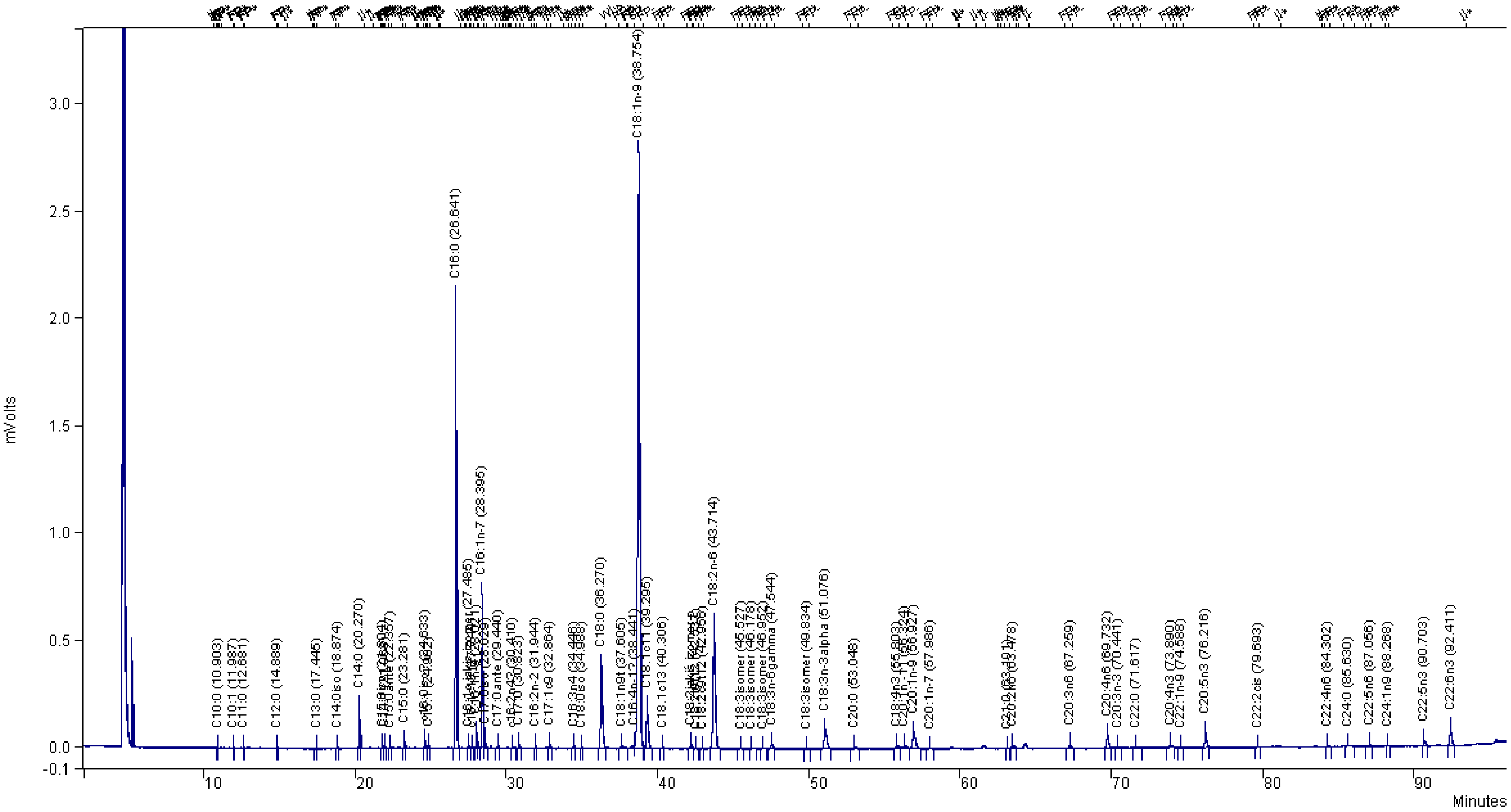

Figure S6. Chromatogram of the separation of fatty acid methyl esters in carp fish balls.
